# Social Support Moderates the Impact of Marital Transitions on Depression for Older Adult Women

**DOI:** 10.1093/geroni/igab046.3327

**Published:** 2021-12-17

**Authors:** Julia Tucker, Nicholas Bishop, Kaipeng Wang, Farya Phillips

**Affiliations:** 1 Texas State University, New Braunfels, Texas, United States; 2 Texas State University, San Marcos, Texas, United States; 3 University of Denver, Denver, Colorado, United States; 4 The University of Texas at Austin, Austin, Texas, United States

## Abstract

Given the rapid growth of older Americans and the increased incidence of divorce among this population, it is paramount to identify negative health outcomes following marital transition and investigate the potential protective role of social support. Our study aims to identify relationships between change in depression and marital transitions, test whether social support moderates this association, and to examine variation by gender. The sample included 3,705 participants from the Health and Retirement Study, who reported being married or partnered in 2012. Changes in marital status were measured between 2012 and 2014 (remained married/partnered (reference), divorced/separated, and widowed). Depression was measured using the Center for Epidemiological Studies Depression short form (CESD-8). Three types of social support from family, friends, and children were assessed: social support, social strain, and social contact. Autoregressive multiple regression was used to examine the relationship between change in depression, marital transitions, social support, and gender. Widowhood and social strain were independently associated with an increase in CESD-8 scores between 2012 and 2014. Significant interactions between social support and social strain, and separation/divorce were identified, and the relationship between social support, depression, and divorce varied by gender. Change in depression was positively associated with social support for separated/divorced females, but not separated/divorced males. These results indicate that social support may modify the influence of divorce on changes in depression among recently divorced older females. These findings can help mental health service providers more effectively target older adults at the greatest risk of depression after experiencing a marital transition.

